# HIV Drug-Resistant Patient Information Management, Analysis, and Interpretation

**DOI:** 10.2196/resprot.1930

**Published:** 2012-06-07

**Authors:** Yashik Singh, Maurice Mars

**Affiliations:** 1 Department of TeleHealthNelson R Mandela school of MedicineUniversity of KwaZulu-NatalDurbanSouth Africa

**Keywords:** Medical Informatics, Bioinformatics, HIV drug resistance, Machine Learning

## Abstract

**Introduction:**

The science of information systems, management, and interpretation plays an important part in the continuity of care of patients. This is becoming more evident in the treatment of human immunodeficiency virus (HIV) and acquired immune deficiency syndrome (AIDS), the leading cause of death in sub-Saharan Africa. The high replication rates, selective pressure, and initial infection by resistant strains of HIV infer that drug resistance will inevitably become an important health care concern. This paper describes proposed research with the aim of developing a physician-administered, artiﬁcial intelligence-based decision support system tool to facilitate the management of patients on antiretroviral therapy.

**Methods:**

This tool will consist of (1) an artificial intelligence computer program that will determine HIV drug resistance information from genomic analysis; (2) a machine-learning algorithm that can predict future CD_4 _count information given a genomic sequence; and (3) the integration of these tools into an electronic medical record for storage and management.

**Conclusion:**

The aim of the project is to create an electronic tool that assists clinicians in managing and interpreting patient information in order to determine the optimal therapy for drug-resistant HIV patients.

## Introduction

The current trend in patient health care is personalized medicine where treatment is individualized, rather than a response to set physical presentations. Thus, access to and interpretation of personal patient information is vital in order to provide a sustainable and useful medical service. The science of information systems, management, and interpretation plays an important role in the continuity of care of patients. This is becoming more evident in the treatment of human immunodeficiency virus (HIV) and acquired immune deficiency syndrome (AIDS). This paper describes proposed research where the aim is to develop a physician-administered artiﬁcial intelligence-based decision support system tool that will facilitate the management of patients on antiretroviral therapy.

The enveloped human immunodeficiency virus infects and destroys the human immune system over a long period of time [1]. The two known strains of HIV are HIV-1 and HIV-2. The rate of replication and infection of the HIV-2 is substantially slower than that of the HIV-1 and accounts for 95% of all HIV infections [2]. HIV-1 is subdivided into four groups representing four separate introductions of simian immunodeficiency virus into humans:

1. Group M is the major HIV-1 group with respect to prevalence (the number of people infected) and incidence (the number of new infections) of the virus;

2. Group O is the outlier group and is mostly restricted to west-central Africa;

3. Group N was discovered in 1998 in Cameroon and is extremely rare; and

4. Group P is a strain closely resembling the gorilla simian immunodeficiency virus.

Currently, Group M is subdivided into nine subtypes or clades—A, B, C, D, F, G, H, J, and K—based on variations in genetic sequence characteristics [3]. However, it is possible for viruses from different subtypes to form mosaic genomes called circulation recombinant forms (CRF). In sub-Saharan Africa, HIV/AIDS is the leading cause of death [4] and it is one of the fastest growing epidemics in South Africa [5-8], where currently there are 5.7million confirmed cases of HIV/AIDS [9]. Demographic information on confirmed HIV-infected patients in South Africa is presented in Table 1.

**Table 1:** Estimated HIV prevalence rates in South Africa [9].

	Women	Men	Total population		
Age range	20–64	20–64	15–46	20–64	All ages
% with HIV	18.1	17.7	18.8	17.9	11.1

HIV infection can be effectively managed with antiretroviral (ARV) drugs, usually in the form of highly active antiretroviral therapy (HAART), which is comprised of a regimen of three drugs from at least two of the following five drug classes [10-13]: reverse transcriptase inhibitors (RTI), nucleoside reverse transcriptase inhibitors (NRTI), protease inhibitors (PI), integrase inhibitors (II), and fusion inhibitors (FI).

Factors that influence treatment of HIV/AIDS with ARVs include poor treatment regimen prescribed by the physician; the World Health Organization (WHO) stage of the disease, which is related to the progression of the disease; levels of plasma drug concentration achieved; how strictly the patient adheres to the regimen; drug resistance [14]; and toxic effects of the drug. Drug resistance is the most critical aspect of treatment. Three common reasons leading to the development of HIV antiretroviral drug resistance are high replication rates, selective pressure, and initial infection by resistant strains of HIV. Thus, it is inevitable that drug resistance will become a reality in most patients’ treatment.

Preventative measures must be taken in order to develop infrastructure that will aid in the management of drug-resistant HIV patients. It is essential to develop techniques that will extract valuable information from little patient data. There must be a means developed to manage, analyze, and interpret patient data.

The aim of this study is to develop a physician-administrated information system that facilitates the clinical management of HIV-positive patients on antiretroviral therapy. This system should be Web-based, patient centric, ascribe to the principles of personal medicine, promote complete health management, and incorporate continuity of care. Creation of this tool will involve:

- Development of an artificial intelligence computer algorithm that analyzes HIV drug resistance data and provides singular interpretable information for a physician indicating which ARVs a patient will be resistant to;
- Investigation of the application of a computer algorithm to predict current and future CD_4_ lymphocyte cell count information given a genomic sequence and other data;
- Integration of the above tools with an electronic medical record system such that it facilitates the storage, acquisition, and management of patient information; and
- Development of a Web-based electronic tool that assists clinicians in determining the optimal therapy for drug-resistant HIV patients.

### Background

#### Medical Informatics

The appropriate application of computer science and associated technology has extended medical care beyond traditional diagnosis and patient management, resulting in extensive cost efficiencies and improved public health outcomes [15]. Areas of medical informatics application include patient records, practice management, clinical measurements, patient education, prescription writing, Web/database resources, clinical records, data collection, clinical decision support, and clinical measurements. Recently, there has been an intentional move towards investigating the synergy between medical informatics and bioinformatics [9]. Bioinformatics is the application of computer science techniques to study how information is represented and transmitted in biological systems starting at the molecular level [16].

The application of genomes in medicine has altered many aspects of medicine. Genome analysis that enhances clinical practice has been successfully applied to asthma [17], cancer [18-20], diabetes [21], and cardiovascular disease [22].

#### HIV Drug Resistance Prediction Algorithms

Testing for HIV drug resistance may consist of wet or dry chemistry laboratory tests, or by employing electronic computerized algorithms [23]. The use of computer algorithms falls under the field of medical informatics. Computer based interpretation algorithms using genomes can also be used to predict HIV drug resistance.

These interpretation algorithms can be generally divided into one of two groups:

- Those based on known domain knowledge (ie, they are based on the fact that certain combinations of known genome mutations cause unequivocal resistance), and
- Those not based on predefined domain knowledge. These algorithms include machine learning and statistical methods.

##### Interpretation Algorithms Based on Domain Knowledge

Domain knowledge interpretation algorithms are based on scientific and published interactions between certain mutations and/or combination of mutations with resistance. This means that all computational decisions concerning resistance are based on known mutation-resistance rules found in published scientific literature. REGA, Agence Nationale de Recherches sur le SIDA (ANRS), and Stanford’s HIVdb algorithm [24] are three examples of well-known domain knowledge interpretation algorithms. These algorithms are widely used in the field and are regarded as gold standards.

REGA and ANRS classify ARV resistance according to three levels: susceptible, intermediate, and resistant. “Susceptible” indicates that a particular ARV drug will be effective against HIV; “intermediate” indicates that the ARV drug is partially effective; and if the ARV is not effective at all, it is classified as “resistant.” HIVdb classifies HIV drug resistance according to five levels: susceptible, potential low-level resistance, low-level resistance, intermediate resistance, and high-level resistance. These algorithms employ Boolean-based rules, some with penalties, and predict resistance by determining which mutations are present and/or absent.

Two other domain-based algorithms are the Drug Resistance SEQuence ANalyzer (DR_SEQAN) and RetroGram. DR_SEQAN was coded by the Universidad Autónoma de Madrid for a Windows environment using Visual Basic. DR_SEQAN classifies three levels of resistance: high-level resistance, increased susceptibility, and no resistance. RetroGram was developed by *Infer*Med Ltd (London, UK) and is built using Arezzo and PROf*orma*. RetroGram generates a suitability ranking for ARV drugs using expert rules. Table 2 describes the accuracy of predicting drug resistance of some algorithms based on domain knowledge.

**Table 2:** Accuracy of predicting ARV drug resistance by the domain-based algorithms, Drug Resistance SEQuence ANalyzer (DR_SEQAN), RetroGram, REGA, and HIVdb [28].

Drug	Accuracy of algorithm (%)			
	DR_SEQAN	RetroGram	REGA	HIVdb
Indinavir	70.8	70.0	84.0	78.0
Nelﬁnavir	66.1	70.5	77.7	73.7
Lopinavir	85.7	88.1	81.0	69.0
Lamivudine	83.1	79.7	78.0	78.0
Zidovudine	79.2	64.6	72.9	68.8
Stavudine	60.0	38.8	67.1	37.6
Didanosine	93.3	20.2	27.9	27.8
Nevirapine	87.9	65.2	72.7	90.9

##### Interpretation Algorithms Not Based on Known Domain Knowledge

Many different pattern recognition and machine-learning algorithms have been applied to find a predictable correlation between genotypic and phenotypic data (called “virtual phenotyping”) [25]. Machine learning may be used to develop a model that predicts virological response. Machine learning is an artificial intelligence computer science technique that tries to find a mathematical model that maps between inputs and outputs of a domain problem.

Virtual phenotyping is growing in popularity. Kuritzkes supports virtual phenotyping as a tool for interpreting viral genotypes [26]. The following are some of the algorithms that have been used:

- Least absolute shrinkage and selection operator (LASSO)
- Ridge regression
- Neural networks, such as multilayer perceptron (MLP) and radial basis neural networks (RBNN)
- Principle component analysis
- Support vector machines (SVM)
- Linear regression models
- Hidden Markov models
- Decision trees
- Multiple correspondence analysis
- Associative classifiers
- k-nearest neighbor algorithm (kNN)

Results produced by these interpretation algorithms are shown in Tables 3–5. These interpretation algorithms have achieved various levels of success, but there are shortcomings in some of the current versions [27]:

- Resistance is interpreted separately for each drug even though therapy consists of combination therapy;
- There is a general lack of data, especially for non-B HIV-1 subtypes;
- Rule-based interpretation is based on the algorithm creator’s knowledge;
- Interpretation algorithms are not always updated even though HIV drug resistance is a rapidly evolving field; and
- Other factors that contribute to treatment failure are not taken into account, such as treatment history, resistance history, viral load history, CD_4_ count history, or plasma drug concentrations.

**Table 3:** Accuracy of predicting ARV drug resistance using the interpretation algorithms, support vector machines (SVM), multilayer perceptrons (MLP), and radial basis neural networks (RBNN) [29].

Drug	Accuracy of algorithm (%)				
	SVM (Energy)^a^	SVM (DEnergy)^a^	MLP (Energy)^a^	MLP (DEnergy)^a^	RBNN
Indinavir	92.6	88.6	87.0	82.5	92.5
Nelﬁnavir	84.9	80.1	86.6	87.1	94.1
Lopinavir	88.6	82.4	92.3	87.9	94.4

^a ^See Bonet et al [29] for detailed information about Energy and DEnergy

**Table 4:** Accuracy of predicting ARV drug resistance using k-nearest neighbor (kNN), decision tree [30], and associative classifier [31] algorithms.

Drug	Accuracy of algorithm (%)		
	kNN	Decision tree	Associative classifier
Indinavir	73.6	93.9	93.0
Nelﬁnavir	81.1	89.5	92.1
Lopinavir	80.8	82.5	89.6

**Table 5:** Accuracy of predicting ARV drug resistance (%) or correlation coefficient (*r*) reported for various other algorithms and machine-learning techniques.

Algorithm	Accuracy (%)	Correlation coefficient (*r*)
HIVdb [32]	84.3	n/a
Visible Genetics/Bayer Diagnostics Guidelines 6.0 [32]	86.3	n/a
AntiRetroScan [32]	89.4	n/a
Committee of neural networks [33]	78.0	n/a
Geno2Pheno [34]	n/a	.6

These shortcomings have led to the creation of many different interpretation algorithms, which produce different resistance measures even if applied to the same resistance profile. These differences are because the studies each used different datasets, subtypes, analysis on drug-naive and drug-experienced patients, and so forth. Conclusions of some studies that reported on the discrepancy of the interpretation algorithms are shown in Table 6.

**Table:** Summary of discrepancies reported using various interpretation algorithms.

Study	Comments
Ravela et al [23]	Studied 4 interpretation algorithms (ANRS-3-02, TRUGENE VGI-6, REGA 5.5, and HIVdb-8-02) and concluded that there was a discrepancy in interpretations in 33% of all resistance profiles tested. The most discordant were NRTIs.
Snoeck et al [35]	Confirmed that there are discordances between the algorithms tested. Suggested it may be due to subtypes.
Vergne et al [36]	Confirmed discrepancies and attributed it to the application of the interpretation algorithms to drug-naive or drug-experienced patients.
De Luca et al (2003) [37]	Concluded that discrepancies in the interpretation algorithms may influence the use of resistance testing over virological outcomes.
De Luca et al (2004) [38]	Studied the application of 13 interpretation algorithms of drug-naive patients and concluded that there are discordances.
Vercauteren and Vandamme [27]	Determined that there is a high level of discordance between the interpretation of NRTI resistance. Also suggests that there should be a “standardization of unique interpretative rules.”
Poonpiriya et al [39]	Indicated that there are discrepancies in the 7 interpretation algorithms they studied.

Collation and interpretation of the contradictory outputs of these algorithms is difficult for physicians treating complex drug-resistant HIV cases, as information is only valuable when it is presented in a clearly interpretable way.

#### Predicting CD4 Count

HIV can be successfully managed with ARV drugs, but information relating to the progression of HIV is vital. HIV infection may be monitored using laboratory [40,41] and clinical marker information [42,43]. Information about a patient’s CD_4 _lymphocyte cell counts are the most widely used data for HIV progression and is recognized as a standard measure of immunodeficiency in HIV-positive patients [44,45]. Thus, the proper use and analysis of information regarding CD_4 _cell counts is vital in CD_4_-guided treatment of HIV [46].

Although the use of CD_4 _count is part of the standard of care in developing countries, the measurement of CD_4 _count requires many complex and expensive flow cytometric procedures, which burden the minimal resources available [45]. The ability to predict current CD_4 _cell count will aid in easing the burden on these resources. A physician may use an electronic tool to economically determine an approximate CD_4 _cell count. If the predicted count is low or indicates that a change in treatment is required, then the physician might order the more expensive laboratory procedure to determine the exact CD_4 _cell count. The ability to obtain information about future CD_4 _count changes will have many benefits to physicians. For example, it will facilitate definite treatment actions, such as changing the regimen in order to prevent opportunistic infection (eg, pneumocystis pneumonia) and delay the onset of AIDS.

Neural network machine-learning algorithms have been used to predict viral load [7,47]. Altmann et al [48] created a machine-learning algorithm that predicts success or failure of therapy, based on viral load, with 80% success. This was later changed by predicting the probability of treatment success based on a degree of predicted HIV drug resistance [49]. However, there is not a chemical test or computer model developed yet to forecast changes to the CD_4 _count.

#### Decision Support System Tool for Managing Therapy

Although models have been created to choose treatment regimens, very few are available in the public domain and/or are easily accessed through a graphical human (user) interface. Currently, there are Web portals that allow one to determine some aspects of HIV drug resistance treatment. These information portals allow one to determine the current HIV resistance profile, graph trends in viral and CD_4 _counts with basic alerts, or store basic patient information. BioAfrica (www.bioafrica.net) is an African-based bioinformatics resource [50]. BioAfrica contains bioinformatics resources that can perform sequence alignments, epitope analysis, tools for proteomics, subtyping and virus genotyping, an RNA virus database, and an HIV drug resistance database and tools. The HIV drug resistance database and tools section is based on the REGA collaborative mode and the Calibrated Population Resistance Tool (CPT). REGA is a drug resistance database developed by the REGA Institute, MyBioData Biomedical IT Solutions, and the Katholieke Universiteit Leuven. It contains interpretation algorithms and stores some clinical data related to HIV treatment. CPT was developed at Stanford University and determines the prevalence of HIV drug resistance in a population.

Some of the other international Web portals for managing HIV treatment information are listed in Table 7.

**Table 7:** Descriptions of Web portals for managing HIV treatment information.

Web portal	Description
Stanford University HIV Drug Resistance Database [51]	This portal determines the interpretation results of the REGA Institute rules, Agence Nationale de Recherches sur le SIDA (ANRS) rules and the Stanford HIVdb rules. It also allows the use of specific user-defined rules using the Algorithm Specification Interface (ASI) and also allows one the opportunity to create a graphical record of a patient’s ARV history, viral loads, CD_4 _counts, and sequence data.
HIVResistanceWeb [52]	This information portal allows information sharing on ARV resistance and clinical virology. It has a store and forward email-based system that allows one to interact with experts.
Los Alamos HIV database [53]	This information portal contains data on genomes, epitopes, drug resistance mutations, and vaccine trials. It is funded by the Division of AIDS of the National Institute of Allergy and Infectious Diseases (NIAID).

These individual information portals are limited by the following:

- The tools they employ in determining HIV drug resistance information. Each information portal uses its own interpretation algorithm and, if collaboration does exist, it consists of simply reporting the outputs of the various algorithms. This causes confusion as some of these interpretation algorithms are disparate, even when the same mutations are analyzed.
- They do not have any means of a real-time expert consultation.
- They are not integrated into a full electronic medical record, which will add the advantage of continuity of care and facilitate tele-HIV-management.
- No individual portal has a variety of tools that can be used to manage HIV therapy.

## Methods

### Part 1: Developing a Single Interpretation Algorithm

The goal of Part 1 is to develop an HIV drug resistance interpretation algorithm capable of providing a single interpretation to genomic analysis.

This part of the study is divided into three main objectives: (1) determining the extent of the disparate information provided by some gold standard interpretation algorithms using the latest version of the interpretation algorithms; (2) developing a novel algorithm to collate the HIV drug resistance interpretation information of these gold standard algorithms into a single easily understandable output; and (3) analyzing the collated algorithm in terms of specificity, sensitivity, and accuracy.

1. Determining the extent of the disparate nature of some gold standard interpretation algorithms using the latest version of these algorithms.

Over time with each new version, interpretation algorithms have improved in predicting ARV drug resistance. Previous comparisons between interpretation algorithms have had some shortcomings:

- Each interpretation algorithm has different measures or levels of resistance;
- Non-contemporary versions of interpretation algorithms were used in the interpretation;
- The interpretation algorithms were applied to different data sets; and
- Few interpretations make use of complex statistical analysis to determine if the differences are in fact significant or not.

The latest versions of different interpretation algorithms will be applied to a single data set extracted from a publicly available anonymized database, the Stanford HIV drug resistance database [51]. The measures of resistance for each interpretation algorithm will be determined, grouped, and analyzed.

2. Developing a novel algorithm to collate the HIV drug resistance interpretation of these gold standard interpretation algorithms into a single output.

The gold standard algorithms may be collated by:

- Weighted output. Different levels of complexity may be applied to determine a single interpretation from multiple interpretations. A simple majority-voting scheme may be applied, where a count of the interpretations of each algorithm is kept. The single interpretation is obtained by determining the resistance outcome with the highest weighting.
- Machine learning on gold standard outputs. Different machine-learning techniques may be applied to the data in order to obtain a single interpretation. Machine-learning techniques work by determining a mapping between a given set of input and desired outcomes and then, using this learnt mapping function, it predicts the output, given a set of inputs. Each interpretation produced for a single resistance profile by the different interpretation algorithms can be the input to a machine-learning algorithm. The output will be the actual HIV-ARV resistance measure determined by fold resistance values. The algorithm will then learn a mapping between the interpretation results obtained using various interpretation algorithms and the actual HIV-ARV resistance measure. One such algorithm that may be employed is a support vector machine.
- Creating a simulated boosted dataset both by modeling the strengths and weaknesses of the gold standards.

3. Analyzing the collated algorithm in terms of specificity, sensitivity, and accuracy.

The specificity, sensitivity, and accuracy associated with predicting ARV drug resistance will be calculated for each algorithm and then compared using statistical analysis.

#### Contribution

The literature does not indicate the current state of disparity between gold standard interpretation algorithms. Combining the interpretation algorithms to form one single interpretation is novel.

### Part 2: Predicting CD4 Count From Genome Data

This part of the study may be divided into three parts:

1. Investigating the possibility of creating a machine-learning algorithm that predicts the current CD_4_ count of a patient using genome sequences, viral loads, and time;
2. Investigating the possibility of creating a machine-learning algorithm that forecasts the medium term change in CD_4_ count of a patient using current genome sequence;
3. It is acknowledged that genome sequencing is more expensive and resource intensive than CD_4_ cell count measurement. However, the cost of genome sequencing is offset by the numerous bioinformatics applications that may be applied to the genome sequence to predict and analyze other physiological measurements and diseases. This study, however, will also investigate the possibility of creating a machine-learning algorithm that forecasts the medium term change in CD_4_ count of a patient using standard of care data.

#### Methods

Datasets will be obtained from the Stanford HIV drug resistance database (http://hivdb.stanford.edu/), which is publically available and contains data from clinical trials. Subtype B consensus protease (PR) genome sequences, CD_4 _count, viral load, and the number of weeks from the baseline measure of CD_4 _count for each patient sample will be determined by joining individual datasets using the sample identifier (the unique number that identifies a sample) and date. Data of patient’s genome sequences and associated viral load and CD_4 _count data at different time points will be extracted.

The changes in CD_4 _count will be grouped into categories and a classification model will be built based on the changes. Different groups of inputs will be created and each will feed into the machine-learning algorithm separately, forming three models. Some of these input groups will be:

- Input1: consisting only of genome sequence;
- Input2: consisting of genome sequence and current viral load; and
- Input3: consisting of genome sequence, current viral load, and number of weeks from the current CD_4_ count to baseline CD_4_ count.

#### Contribution

Currently, there is not a chemical test or computer model developed to forecast future changes to the CD_4 _count.

### Part 3: Developing Web-based Tool for Determining Optimal Therapy

The goal of Part 3 is to develop a Web-based electronic tool that assists clinicians in determining the optimal therapy for patients indicative of HIV drug resistance.

There is evidence that suggests that resistance testing is beneficial:

- A two-factorial (genotyping and expert advice), randomized, open label, multicenter trial [54] was undertaken to determine if there is any benefit in using genotyping rather than the expert’s direct knowledge when prescribing ARVs. The conclusion was that genotyping benefits the overall optimal care of HIV patients.
- The VIRalliance SAS [55] group clearly demonstrated in their study that “resistance testing prior to initiating or switching antiretroviral therapy” is essential.
- Mascolini et al [56] questioned 600 clinicians about the effect of resistance testing on their diagnosis and regimen they prescribe. They confirmed that “if the assay detected partly or multidrug-resistant virus, then the large proportions of respondents (indicated that they would) change their treatment choice.”
- Hirsch et al [57] found that “resistance testing can improve virological outcome among HIV-infected individuals.”
- The Can Resistance Enhance Selection of Treatment (CREST) [25] study (a 48-week follow-up randomized trial) found that genotypic drug resistance testing may be beneficial in the management of HIV infection.

#### Objective

The goal is to combine the tools mentioned previously, and possibly other bioinformatics tools, into one seamless application.

#### Methods

Java, HyperText Markup Language (HTML), PHP: Hypertext Preprocessor (PHP), and other paradigms will be used to create a Web-based portal that will integrate the different tools. An important aspect to take into account when building the model for the HIV management system is security. Dwivedi et al [58] argued that electronic medical records will only become a reality if security takes a prominent role in design considerations and during implementation. Two of the most promising techniques for incorporating security into any information system are public key infrastructure and biometrics. Public key encryption is a nondeterministic polynomial time complex technique that ensures high-level security. Biometrics use physical of behavioral traits to identify an individual. The exact means of integration and security model to be used will only be determined after the individual tools are built.

#### Contribution

The creation of an electronic medical record-based virtual HIV clinical support system that aids in the determination of the best HAART combination, using a combined ARV resistance interpretation, CD_4 _count prediction, and the other methods described is novel.

## Conclusion

The outcome of this study is to facilitate the acquisition, storage, management, analysis, and interpretation of information by physicians. In personalized medicine, it is essential that information be interpreted and presented clearly and concisely. We expect that the proposed tool will aid in this aspect.

## Abbreviations

AIDS: acquired immune deficiency syndrome
ANRS: Agence Nationale de Recherches sur le SIDA
ARV: antiretroviral
ASI: algorithm specification interface
CPT: Calibrated Population Resistance Tool
CREST: Can Resistance Enhance Selection of Treatment
CRF: circulation recombinant forms
DR_SEQAN: Drug Resistance SEQuence ANalyzer
FI: fusion inhibitors
HAART: highly active antiretroviral therapy
HIV: human immunodeficiency virus
kNN: k-nearest neighbor algorithm
LASSO: least absolute shrinkage and selection operator
MLP: multilayer perceptron
NIAID: National Institute of Allergy and Infectious Diseases
NRTI: nucleoside reverse transcriptase inhibitors
PHP: PHP: Hypertext Preprocessor.
PI: protease inhibitors
RBNN: radial basis neural networks
RTI: reverse transcriptase inhibitors
SVM: support vector machines
WHO: World Health Organization
